# Safe Plastic Surgery of the Breast II: Saving Nipple Sensation

**Published:** 2017-11-21

**Authors:** Steven Schulz, Matthew R. Zeiderman, J. Stephen Gunn, Charles A. Riccio, Saeed Chowdhry, Ronald Brooks, Joshua H. Choo, Bradon J. Wilhelmi

**Affiliations:** ^a^The Ohio State University Department of Plastic Surgery, Columbus, OH; ^b^Division of Plastic and Reconstructive Surgery, Hiram C. Polk Jr. M.D. Department of Surgery, University of Louisville School of Medicine, Louisville, Ky; ^c^Department of Plastic Surgery, University of California Irvine; ^d^Department of Plastic Surgery, University of Tennessee Memphis; ^e^Chicago Medical School, Rosalind Franklin University of Medicine and Science, Chicago, Ill; ^f^University of Southern Alabama Plastic and Reconstructive Surgery, Mobile

**Keywords:** nipple innervation, reduction mammoplasty, nipple, breast reconstruction, nipple-areola complex

## Abstract

**Background:** Since its inception, reduction mammoplasty has matured considerably. Primary evolution in clinical research and practice initially focused on developing techniques to preserve tissue viability; breast parenchyma, skin, and nipple tissue that has expanded to include sensation and erectile function play a large role in the physical intimacy of women. Studies regarding primary innervation to the nipple are few and often contradictory. Our past anatomical study demonstrated that primary innervation to the nipple to come from the lateral branch of the fourth intercostal nerve. We propose an unsafe zone in which dissection during reduction mammoplasty ought to be avoided to preserve nipple sensation. **Objective:** To identify the trajectory of innervation to the nipple and translate these findings to the clinical setting so as to preserve nipple sensation**. Methods:** Eighty-six patients underwent reduction mammoplasty using the Wise pattern inferior pedicle (n = 72), vertical Hall-Findlay superomedial pedicle (n = 11), and Drape pattern inferior pedicle (n = 3). Aggressive dissection in the most superficial and deep tissue in the inferolateral quadrant of the breast was avoided. **Results:** All 86 patients reported having the same normal sensation to the breast at postoperative evaluation. **Conclusions:** The fourth intercostal nerve provides the major innervation to the nipple-areola complex. Avoiding dissection in inferolateral quadrant “unsafe zone” of the breast during reduction mammoplasty can reliably spare nipple sensation and maximize patient outcomes.

Since its inception, reduction mammoplasty has matured considerably. Primary evolution in clinical research and practice has focused on developing techniques to preserve tissue viability, breast parenchyma, skin, and nipple tissue. Previously, women with macromastia were believed to be more concerned with breast size and shape over mammary sensation. Presumably, the improved aesthetic result led to an improved body image and enhanced feelings of sensuality. However, surgery today involves not only preserving tissue viability but also function in terms of sensation. The nipple serves as a sensate unit in erectile function and plays a large role in women's physical intimacy. Nipple sensation has shown to be a valuable part of women's psychological and sexual health. While preservation of nipple sensation is of utmost importance, the literature regarding primary innervation of the nipple is scant and contradictory.^1^^-^5 To strengthen the literature on this topic, the authors reviewed current literature on nipple innervation, performed additional anatomical studies,^6^ and applied their findings to the clinical setting to identify a safe zone for reduction mammoplasty to preserve nipple sensation.

## METHODS

Findings from a previously conducted anatomical study that identified an “unsafe zone” for reduction mammoplasty in the inferolateral quadrant of the breast were applied to the clinical setting.^6^ Special care was taken to avoid dissection in this area. Specifically, dissection of the superficial tissue 1 to 2 cm deep to the dermis and tissue running 1 to 2 cm superficial to the pectoralis major was avoided in the lateral pillar of breast tissue of the 86 patients who underwent reduction mammoplasty using the Wise, Drape, and Hall-Findlay techniques. Seventy-six patients underwent bilateral reduction mammoplasty and 10 underwent unilateral reduction mammoplasty. Seventy-two patients had a Wise pattern inferior pedicle reduction.

All patients are marked preoperatively with nipple placed at the level of the inframammary fold along the breast meridian. A Wise pattern skin excision is designed utilizing 7- to 8-cm limbs of a triangle, with the apex being the new position of the nipple. That distance represents the new desired nipple to inframammary fold distance that will be created after completion of the reduction. In this technique, a 10 cm wide inferior dermal pedicle is utilized, with 8 cm being medial to the breast meridian and 2 cm being lateral to the meridian. A Wise pattern skin excision is accomplished, with the majority of the reduction being accomplished medially and laterally taking care to preserve tissue over the pectoralis and serratus fascia in the unsafe zone. Mastectomy flaps are dissected with a thickness of 2 cm to ensure adequate blood supply to the flaps. A small amount of additional breast parenchyma is kept on the pedicle superior to the nipple-areola complex (NAC) in order to create more upper-pole fullness after the inset of the nipple and closure. The pedicle is plicated with 3 to 4 interrupted sutures to reset the length of the pedicle to the same length of the preoperative nipple to inframammary fold distance. This maneuver not only helps with inferior pole contouring and controlling the inferior breast but also places the NAC at the level that was determined by the preoperative measurements. Closure is accomplished with a deep dermal layer starting medially and laterally to the converging point of the flaps at the breast meridian. Any tissue redundancy is preferentially brought into the median of the breast to remove any medial and lateral dog ears. A running 3-0 PDS is then utilized to close the new inframammary fold. The vertical incision is closed in a similar fashion with a deep layer of 2-0 and running 3-0 PDS. Then 1.5 cm is measured in each direction of the apex of the vertical limb, which represents the diameter of the new NAC. This is excised, and because of the unequal distribution created by the procedure, this usually is not a perfect circle and some minor additional skin excision is usually needed to create perfect circular opening for the inset of the nipple. The inferior pedicle and the NAC are right underneath the flaps at this location due to the previous plication sutures that were performed in the pedicle. The nipple is inset with a deep layer of 3-0 PDS and a running purse string 4-0 Monocryl to ensure the circular shape of the NAC. Drains are occasionally utilized in patients requiring 1000 g or more in reduced volume to assist with fluid evacuation and management of hematomas.

Three patients underwent inferior pedicle Drape pattern reduction with no vertical scar. Eleven patients had a Hall-Findlay vertical reduction with superomedial pedicle. Nipple sensation was reported by subjective patient evaluation at 6-month postoperative follow-up.

## RESULTS

**Figure 1:**
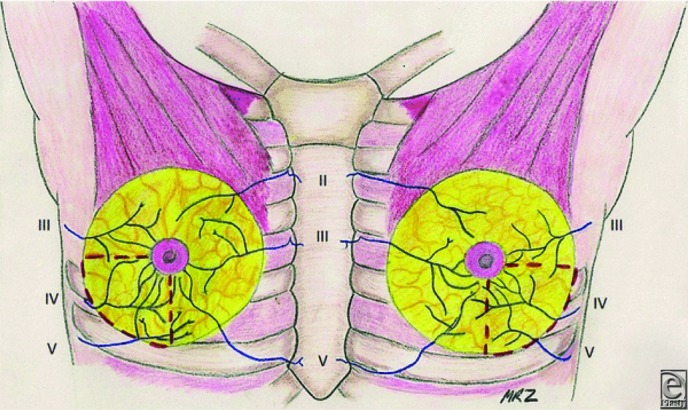
Anterior view of intercostal nerve innervation to the nipple. The red dashed lines demarcate the inferolateral breast quadrant to be avoided during surgical dissection so as to preserve nipple sensation. Reprinted with permission of MR Zeiderman and CA Riccio et al.

**Figure 2:**
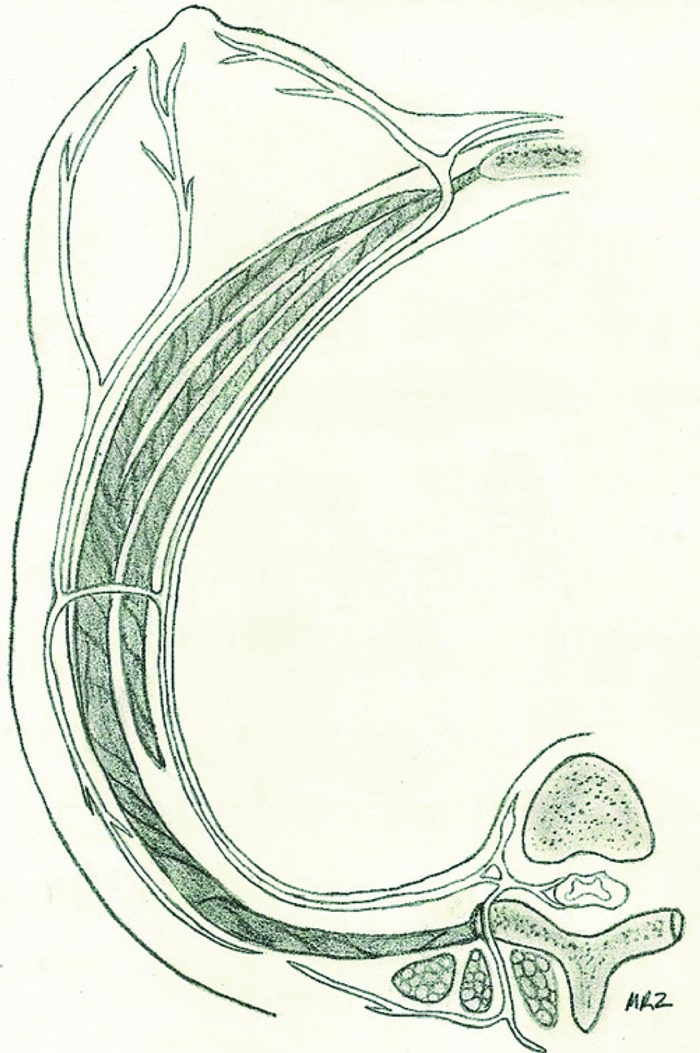
Cross-sectional illustration of intercostal nerve innervation to the nipple. Reprinted with permission of MR Zeiderman and CA Riccio et al.

**Figure 3:**
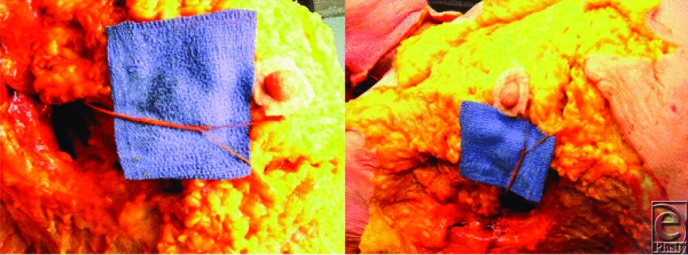
Photographs from cadaveric dissection, highlighting the course of the fourth intercostal nerve in the inferolateral quadrant.

**Figure 4:**
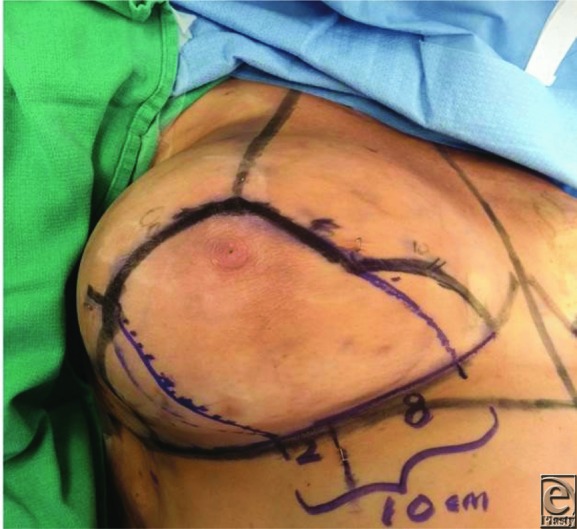
Right breast markings demonstrating the 10 cm inferior dermal pedicle with 8 cm medial to the breast meridian and 2 cm laterally.

**Figure 5:**
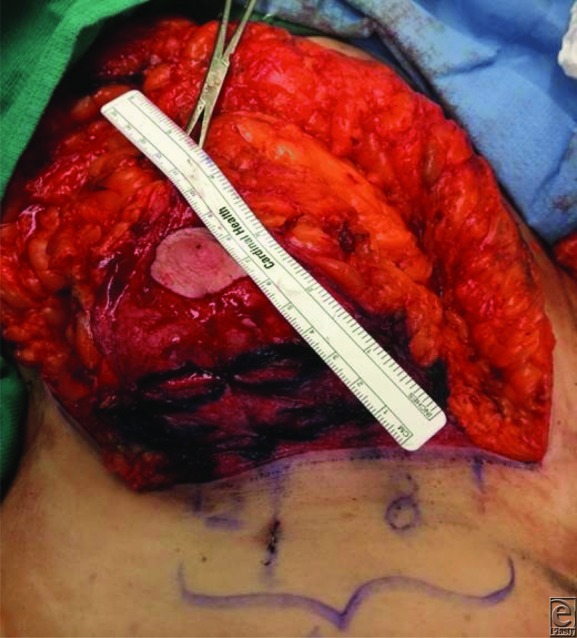
The inferior dermal pedicle after plication.

Additional investigation of our previously published anatomical results affirms 3 to 5 branches of the fourth intercostal nerve to primarily innervate the nipple on 24 of 30 breast dissections. Four breasts received primary innervation from the third intercostal nerve and 2 from the fifth intercostal nerve. In half the specimens, accessory innervation from the third and fifth intercostal nerves provided medial branches to the nipple (Table 1). On the left side, the nerve travels toward the nipple at the 4 o'clock position, whereas it enters at the 8 o'clock position on the right side. The nerve pierces the chest fascia above the fifth rib 3 cm lateral to the border of the pectoralis major muscle and travels through the gland approximately 0.5 to 1 cm above the pectoralis in an inferolateral position toward the nipple. More superficial nerves can be identified running through the gland 0.5 to 1 cm deep to the dermis, also coursing from inferolateral position toward the nipple (Figs 1-3).^6^ Clinical results identified no loss of nipple sensation for all 86 procedures, a total of 162 breasts. Total reduction weights ranged from 350 to 2500 g (Table 2). The average patient age was 43.5 years, and the average total resection weight was 1398.5 g. Average resection weight per breast was 742.4 g. All patients reported normal nipple sensation upon postoperative evaluation. One reoperation was required for wound dehiscence. Four patients had delayed wound healing without need for operative intervention (Figs 4 and 5).

## DISCUSSION

Breast reduction surgery has evolved considerably through the centuries, as has the popularity of procedure; 100,825 breast reductions were performed in the United States alone in 2015 according to the American Society of Plastic Surgeons.^7^ Prior to the late 1800s, breast amputation was the procedure performed to eliminate excessively large breasts. Theodore Galliard-Thomas was the first to advocate preservation of some part of the glandular tissue in the 1880s.^8^^,^^9^ The mid-1920s brought the techniques of Lexar and Kraske to transpose the nipple after creating subcutaneous flaps.^9^ Thorek^10^ was the first to perform a free nipple graft for excessive macromastia. Schwarzman et al^11^ developed the concept of de-epithelialization to maintain the nipple complex on a dermal plexus in the 1960s. Wise^12^ built upon Biesenberger's procedure of separating the skin from the gland and transposing the nipple by developing resection patterns to aid in safer, more reliable reductions.^13^ The vertical bipedicle dermal reduction was popularized later by McKissock.^14^ Inferior pedicle techniques were developed by Robbins,^15^ Courtiss and Goldwyn.^16^ Courtiss^17^ later described using liposuction alone as a reduction method. The vertical reduction was later popularized by Arie,^18^ Lassus,^19^^-^21 Lejour et al,^22^ Hall-Findlay^23^, and modifications by Hoffmann.^24^ Primary goals in the progression of reduction mammoplasty procedures through the years have been tissue viability, shape, contour, and scar aesthetics.

However, many advocate that nipple sensation is paramount to patient satisfaction as well. As the nipple is perhaps the most sensitive area of the breast, it serves a significant role in a woman's sexual life. Erectile function and sensation are frequently necessary for both the woman herself and her partner. Consequently, loss of these functions has a detrimental impact on procedure outcome and patient satisfaction.^25^^-^29 Previous studies have demonstrated that the majority of women feel that nipple-areola sensitivity is an important part of their sexual life; of those women who underwent breast surgery and lost nipple sensation, the majority were significantly bothered by the result.^29^

In general, patients undergoing breast reduction surgery demonstrate high satisfaction due to the improvement in neck, shoulder, and back pain. However, loss of sensation to the nipple results in an inferior outcome. Anatomical analysis and understanding of the innervation of the NAC help guide the plastic surgeon in avoiding damage to the nerves of the nipple. Our anatomical study demonstrates the innervation of the nipple to come laterally from 3 to 5 branches off the fourth intercostal nerve. In addition, in some specimens, the third and fifth intercostal nerves provided accessory innervation. The fourth intercostal nerve pierces the fascia of the fifth rib just lateral to the border of the pectoralis major muscle. The nerve travels to the NAC through the inferolateral position of the NAC. Previous studies have demonstrated the lateral branch of fourth intercostal nerve to be the most reliable innervation to the NAC.^1^^-^5 Other studies also demonstrated accessory innervation of the nipple to come from both anterior and lateral branches of the second through sixth intercostal nerves.^1^^,^^3^ However, not all innervation to the NAC can be reliably salvaged during reduction mammoplasty because of the need for dissection in multiple breast quadrants. Careful dissection in the region of primary innervation to the nipple by avoiding breast tissue resection in either too superficial or too deep of a plane can result in better nipple sensation and patient satisfaction.

The understanding gained from the anatomy laboratory crossed to the clinical setting in preserving the nipple-areola sensation in 86 patients undergoing reduction mammoplasty. Reduction weights ranged from 350 to 2500 g, and both inferior pedicle and vertical/superomedial pedicle techniques were performed and no numbness resulted. Lessons learned in the anatomy laboratory translated to the clinical application, as care was taken to avoid excessive resection and dissection in the inferolateral areas of the breast so as to preserve the innervation of the NAC. A simple adaptation to commonly performed procedures yields improved results. The Wise pattern and Hall-Findlay techniques have gained much popularity in recent decades.^30^ The Wise pattern inferior pedicle is easy to perform and learn and can be used in almost any circumstances.^30^ The Hall-Findlay superior pedicle vertical reduction technique offers the advantages of rapid operative time and satisfactory aesthetic and functional outcomes for appropriately selected patients.^30^ Enhancing functional outcomes increases the utility of these popular techniques.

Frequently, the plastic surgeon must individualize therapy to the patient. A fixed procedure does not always apply to every clinical scenario. Adhering to principles of techniques and knowledge of anatomy frequently serves as a foundation for the reconstructive surgeon when planning procedures. This study can aid the novice and experienced surgeons in obtaining quality outcomes in terms of not only aesthetics but also function.

## CONCLUSION

Preserving nipple sensation is a valuable goal in breast surgery. Many women value nipple sensation as a significant component of sexuality and quality of life. The innervation of the nipple is predictable based on anatomical findings. An unsafe zone can reliably be avoided in the inferolateral area of the breast. Clinical application of these findings demonstrates the possibility to reliably maintain the nipple as an aesthetic and sensate unit.

## Figures and Tables

**Table 1:** Primary and accessory innervation of the nipple*

Specimen	Side	ICN	Accessory ICN	No. of branches
1	L	4	3	3
2	L	4	5	3
3	R	4	5	5
4	R	5	5	5
5	L	4	3	4
6	R	4	3	4
7	R	3	4	4
8	L	4	5	3
9	L	4	5	5
10	R	4	3	3
11	L	4	3	4
12	R	4	3	3
13	L	3	5	5
14	R	4	5	5
15	L	4	3	4
16	R	4	3	5
17	R	5	4	3
18	L	4	5	5
19	R	4	4	4
20	L	4	3	5
21	L	4	3	4
22	R	4	5	3
23	R	4	3	2
24	L	4	3	3
25	R	4	5	3
26	L	4	3	4
27	R	3	4	3
28	L	4	3	3
29	L	4	5	2
30	R	3	4	4

*Specimen data for 30 dissections equally distributed between left and right sides. Twenty-four of 30 showed primary innervation from the fourth ICN. Accessory innervation came from ICN 3-5. The primary nerves have 3 to 5 branches to supply the nipple. ICN indicates intercostal nerve; L, left; and R, right.

**Table 2:** Resection weight data from study population based on technique

Reduction method	Patients, n	Total breast, n	Avg. total resection weight, g	Avg. resection weight/breast, g	Range of individual resection weight, g	SD of individual resection weight, g	SD of total resection weight, g	Complications, n
Drape inferior pedicle	3	5	1695.3	1017.2	525-1376	355.7	1093.9	1.0
Wise pattern inferior pedicle	72	139	1484.9	769.2	290-2640	381.3	780.5	4.0
Vertical superomedial pedicle	11	18	752.2	459.7	334-575	61.1	220.9	0.0
Study	86	162	1398.5	742.4	290-2640	374.3	778.6	5.0
